# Repositioning Chromones for Early Anti-inflammatory Treatment of COVID-19

**DOI:** 10.3389/fphar.2020.00854

**Published:** 2020-06-05

**Authors:** Piero Sestili, Vilberto Stocchi

**Affiliations:** Department of Biomolecular Sciences (DISB), Università degli Studi di Urbino Carlo Bo, Urbino, Italy

**Keywords:** COVID-19, inflammation, lung injury, chromones, mast cells

## Abstract

The COVID-19 pandemic is posing an unprecedented sanitary threat. In the absence of specific vaccines and anti-SARS-CoV-2 drugs, medicines that may assist in tackling the emergency and limiting the high number of fatalities are urgently needed. The repositioning of available drugs to treat COVID-19 is the only and rapid option in the face of the lack of direct antiviral agents and vaccines available. In this light it is important to focus on available drugs, which, based on their pharmacodynamics, could plausibly attenuate viral growth as well as COVID-19’s worst complications. This is the case of chloroquine and tocilizumab which seem to limit virus replication and the severity of interstitial pneumonia, respectively. However, these treatments, particularly those aimed at containing inflammation, are still reserved for the most severe cases. This commentary elaborates on the pharmacological rationale of repositioning the mast cell stabilizer chromones as an adjunctive treatment for SARS‐CoV‐2 infection, and proposes their practical clinical testing as an early, safe, and cost-effective anti-inflammatory intervention in COVID-19 to limit the eventual secondary progression toward life-threatening respiratory complications.

On 11 March 2020, SARS-CoV-2, which first emerged in December 2019 in Wuhan, China, was declared a pandemic by the World Health Organization (WHO). While in China the situation has returned to normalcy, in many European countries, USA, and Brazil the number of SARS-CoV-2 positive cases is still growing, dramatically overwhelming the national health systems’ resilience.

Realistically, the development of an effective SARS‐CoV‐2 vaccine (Patel et al., 2020; Zhang and Liu, 2020) or of highly specific anti-SARS-CoV-2 drugs could take too much time (Harrison, 2020). Hence, rational repositioning of existing medications is the only and rapidly available strategy (Harrison, 2020).

To this regard, some drugs and treatments have been repurposed as possibly active against SARS-Cov-2 (Beigel et al., 2019; Li and De Clercq, 2020). The most promising ones, i.e. the viral RNA-dependent RNA polymerase inhibitor remdesivir, chloroquine, hydroxychloroquine (Gao et al., 2020; Luo et al., 2020), are being evaluated in accelerated clinical trials (Gao et al., 2020). Increased attention is being devoted to convalescent plasma collected from recovered COVID-19 patients, which at this time represents the only available highly-specific and promising antiviral option currently under clinical trials (Franchini et al., 2020).

In parallel, the better comprehension of the pathogenic mechanisms of COVID-19 and their sequences has prompted the use and clinical evaluation of host-directed drugs such as heparin (Porfidia and Pola, 2020) or tocilizumab (Siddiqi and Mehra, 2020).

According to a recent article, the COVID-19 disease can be divided into three escalating phases (Siddiqi and Mehra, 2020). Stage I occurs at the time of inoculation and early establishment of the disease, i.e. when virus multiplication causes mild respiratory and non-specific symptoms (malaise, fever, and a dry cough). Drug-containment of viral replication at this early stage could greatly ameliorate prognosis and recovery. Stage II is characterized by further viral multiplication with localized and increasing inflammation in the lungs. Patients develop a viral pneumonia, with cough, shortness of breath, and fever without (Stage IIA) or with (Stage IIB) hypoxia. Stage IIB usually requires hospitalization. Importantly, transition to the life threatening Stage III corresponds to the onset of a cytokine storm and hyper-inflammation which are both recognized as the key players in the progression toward severe interstitial pneumonia, acute respiratory distress syndrome (ARDS), and coagulopathies (Kowalewski et al., 2020).

In this late phase, corticosteroids in concert with the use of cytokine inhibitors such as tocilizumab are used to reduce local and systemic hyper-inflammation before it results in fatal multi-organ dysfunction (Siddiqi and Mehra, 2020). Preliminary data from case reports (Michot et al., 2020) seems to confirm the efficacy of tocilizumab in Stage III patients, pointing to the importance of the abnormally dysregulated inflammatory response in COVID-19.

The three stages may have a duration of several weeks, but a crucial turning point seems to occur in Stage II when the decrease of viral invasion is paralleled by a progressive increase of inflammation, which is likely prodromal to the exacerbation of Stage III (Siddiqi and Mehra, 2020). Hence, according to this scenario, anti-inflammatory drugs should be given before the presentation of Stage II symptoms (Sheppard et al., 2017). To this end, however, tocilizumab and corticosteroids are questioned, or not recommended in earlier stages because of the cost and sub-optimal safety issues (Elmedany et al., 2019), and because of immunosuppressive activity (Veronese et al., 2020), respectively.

NSAIDs, rather, could be used for the early and mild control of inflammation but, due to a questionable warning on their supposed grave adverse effects in COVID-19 patients (de Girolamo et al., 2020), the only recommended one is paracetamol which, unfortunately, has no anti-inflammatory activity. As a consequence, despite the fact that stage I and IIA are likely crucial for the progression of the malady, there is yet no consensus on an adequate anti-inflammation treatment for these COVID-19 phases.

Therefore, the need for early and more tolerable anti-inflammatory strategies should speed up the repositioning of existing drugs. A potential drug class to fill this gap might be the so called “mast cell (MC) stabilizers” (Sinniah et al., 2017) such as the chromones (CR). Cromoglycate (CG), was the first approved CR for the treatment of asthma and other allergic conditions in the ‘60s. CG was followed by its close analogue nedocromil, and later by structurally unrelated agents such as lodoxamide and ketotifen (Zhang et al., 2016). Interestingly, plant-derived flavonoids (e.g. quercetin and luteolin) are also known to exhibit MCs stabilizing capacity. In addition, other widely prescribed drugs (statins, nifedipine) (Zhang et al., 2016) have been reported to exert some effect on MC degranulation. The main mechanism of action of MC stabilizers consists in the MCs plasma membrane “stabilization” which reduces the typical degranulation and activities of this immune cell type (Sinniah et al., 2017).

MCs represent one of the most important cells in the immune system, playing a pivotal role in the mechanisms underlying the initiation and persistence of the inflammatory response (Gonzalez-de-Olano and Alvarez-Twose, 2018). Although MCs are relatively few as compared with other immune cells, they are present in all human tissues, particularly in those acting as a physical or physiological interface such as the skin, the gastrointestinal and respiratory tracts, and the vasculature endothelium (Gonzalez-de-Olano and Alvarez-Twose, 2018; Varricchi et al., 2019).

The interactions of MCs with viruses and pathogen products are complex and can result in both detrimental and positive responses in the host. The Coronaviruses are rapidly targeted by immune cells including MCs located in the submucosa of the respiratory tract (Kritas et al., 2020). The host response to RNA virus invasion activates Toll-like receptor-3 (TLR3) on MCs which produce interferon (IFN) α and β and IL-8, and recruit NK cells (Meng et al., 2016). Hence, in this light, MCs serve exquisitely as anti-viral immune cells. However, in the opposite direction, the virus also induces sensitization of MCs and the synthesis of IgEs that bind to the FcϵR immunoglobulin receptor and trigger the inflammatory reaction (Gonzalez-de-Olano and Alvarez-Twose, 2018). In the presence of favoring conditions and ongoing alveolar epithelial damage, MCs more likely start acting in a proinflammatory direction (Liebler et al., 1998; Virk et al., 2016). This view has been corroborated by a very recent editorial by Kritas et al. (Kritas et al., 2020) suggesting that MCs play a significant pathogenic role in COVID-19 pneumonia. According to this scenario, virus-stimulated mucosa MCs release cytokines and mediators (TNF-α, IL-1, IL-6, IL-33, IL-18 and proteases, histamine, prostaglandin D2, leukotriene C4) resulting in lung inflammation and bronchoconstriction (Kritas et al., 2020). In such a negative setting MCs plausibly facilitate the transition into COVID-19 Stages IIB and III. To this regard, although it was a completely different experimental setting, it is worth noting that pre- and co-treatment with CG reduced the severity of experimentally induced acute lung injury in rats, an effect ascribed to the drug-induced stabilization of MCs (Zhang et al., 2013).

The pharmacodynamics of CRs is not limited to MC stabilization. Indeed, as comprehensively reviewed by Sinniah et al. (2017) CRs exert a general inhibitory action on multiple cellular processes and biochemical targets, such as polymorphonuclear leukocytes activation and migration, macrophage activation, tachykinin action, adhesion molecule expression, eicosanoid and cytokine release, blockade of chloride channels, inhibition of eicosanoids release, and cytokine production. CRs have also been shown to activate an endogenous anti-inflammatory loop, the Anx-A1/FPR system, which exerts a key ‘anti-inflammatory and pro-resolution’ role in several important host defense responses (D’Acquisto et al., 2008; Perretti and D’Acquisto, 2009).

In a human study, exposure to organic dust induced a significant increase in inflammatory cells and soluble components (neutrophils, IL-6 and TNF-α) in bronchoalveolar and nasal lavage fluid in voluntary subjects; interestingly CG-treatment prevented these effects (Larsson et al., 2001).

Finally CRs reportedly exert direct antioxidant activity (Debbasch et al., 2001; Sadeghi-Hashjin et al., 2002), which may concur to hamper radical-induced tissue damage occurring in viral and SARS-CoV-2 lung injury (Zhang et al., 2020).

Then CRs are capable of triggering a broad range of therapeutically valuable effects which—as for other proposed off-label uses (Sinniah et al., 2017)—could be exploited to treat COVID-19.

Intriguingly, a recent study by Han et al. (Han et al., 2016) on mice infected with the influenza A virus H5N1, showed that animals pre- and co-treated with CG survived significantly better than sham-treated mice. CG-treated mice showed only mild pathological changes with fewer inflammatory cell infiltration in the nose, trachea, and lungs. Moreover, significantly lower expression of IL-6, TNF-α, and TLR-3 was detected in the lungs of CG-treated mice. Since no difference was observed in the virus load between groups, the authors concluded that the CG therapeutic effect in H5N1 virus-infected mice depended on the attenuation of the inflammatory response rather than on the inhibition of viral replication in the lungs. Although the latter is an animal study, the fact that the CG-membrane stabilizing capacity has been questioned in mice (Oka et al., 2012) and the fact that there is no strict relation between H5N1 and SARS-CoV-2, other than that they are both airway-targeting RNA viruses, it seems that CG acts on virus-unrelated targets and could support the concept of using CRs as an early and/or adjunctive anti-inflammatory tool in COVID-19 patients.

Along with the above rationale, CRs repurposing against COVID-19 might gain ground not only because of nearly 50 years of clinical experience in lung diseases but also—unlike the more active corticosteroids and biologics used in disease Stage III—because of its excellent safety profile, its efficacy through the inhalatory route, the absence of significant immunosuppressive activities as in steroids, and, finally, the favorable cost-effectiveness. Notably, with regard to aerosolized CG, a new polysaccharide microparticles formulation significantly ameliorates pulmonary delivery, efficacy, and patient compliance (Gallo et al., 2017). All these features would make CRs valuable candidates for the treatment of COVID-19 patients starting from Stage I. For the sake of completeness, however, CRs also have some pharmaco-kinetics/-dynamics limits consisting of poor absorption through the oral route and a possible induction of rapid tachyphylaxis in an organ/tissue location-dependent manner (Pearce et al., 1989).

An alternative to CRs might be the antihistaminic ketotifen possessing potent MC stabilizing activity and better oral pharmacokinetics, although no clear-cut demonstration of its clinical superiority in the management of asthma has been provided yet (Schwarzer et al., 2004; Zhang et al., 2016).

Along the same line, it is worth noting that recent cell culture-based studies indicate some naturally occurring flavonoids, namely quercetin (Weng et al., 2012), luteolin, and tetramethoxyluteolin (Weng et al., 2015), as having a significant—and even higher as compared to CRs—MCs-stabilizing activity. Furthermore, these polyphenols possess a plethora of biologically relevant capacities including antioxidant (Sestili et al., 1998), anti-inflammatory and antiviral (Theoharides, 2020). Hence, these plant polyphenols—particularly the most active tetramethoxyluteolin (Theoharides, 2020)—might represent a further, mechanistically-coherent nutritional strategy to associate with CRs in a safe and early treatment setting against COVID-19. Unfortunately, unlike CRs, there is poor or no clinical experience with these flavonoids in the treatment of pulmonary conditions and no clear-cut indication regarding their optimal posology.

Although it is currently an inexplicably underrated option, early and timely treatment with drug associations, controlling virus replication and inflammation might allow for modifying the course of disease, improve patients’ recovery rate and time, and treat them in a domestic care setting, avoiding the risk of hospital collapse sadly experienced by most western countries.

Such a large-scale therapeutic strategy would require—along with the implementation of virus testing—a number of available, cheap, safe, and widely diffused drugs hitting COVID-19 from multiple sides. At the moment hydroxychloroquine (Gao et al., 2020) and low MW heparin (Porfidia and Pola, 2020) might fulfill these requisites and be prescribed on a larger scale to control virus replication. From their side CRs (and/or MCs stabilizers) could allow a safe, inexpensive and timely control of airway inflammation, with the aim of anticipating the progression toward life-threatening complications. Along this line, statins are included among the drugs which could be rationally repositioned against COVID-19 for their anti-inflammatory and immune-modulating activity (Phadke and Saunik, 2020): intriguingly, as stated above, statins also exert MC stabilizing effects (Kolawole et al., 2016).

In conclusion, the COVID‐19 pandemic is currently dramatically growing, and drastic actions are urgent for containing its spread and minimizing the death toll. The tentative suggestion to apply CR or MC stabilizers as anti-inflammatory agents prior to the development of acute respiratory complications, remains unproven until tried. The most rapid approach for assessing its feasibility, is to analyze clinical patient records to determine whether there are subjects prescribed with CRs prior to COVID-19 diagnosis, and whether they had better disease outcomes with respect to the general population. Curiously and counter-intuitively, asthma—where CRs are often prescribed—does not seem to be a risk factor for COVID-19 (Lupia et al., 2020). Knowledge gained from such data mining of clinical records, along with the rationale illustrated above, might stimulate the setting of rapid and practical clinical research on the proposed CRs repositioning in COVID-19.

## Data Availability Statement

Publicly available datasets were analyzed in this study. These data can be found here: https://pubmed.ncbi.nlm.nih.gov/.

## Author Contributions

Conceptualization: PS and VS. Writing: PS. Review and editing: PS and VS.

## Funding

The present article has been funded by a grant (recipient PS) from Università degli Studi di Urbino Carlo Bo, Fondi Valorizzazione DISB.

## Conflict of Interest

The authors declare that the research was conducted in the absence of any commercial or financial relationships that could be construed as a potential conflict of interest.
